# Supplementary material for: The Construction and Exploration of a Comprehensive MicroRNA Centered Regulatory Network in Foxtail Millet (Setaria italica L.)
Source: Front Plant Sci. 2022 May 6;13:848474. doi: 10.3389/fpls.2022.848474 (PMC9121102; doi:10.3389/fpls.2022.848474)
Supplement: Supplementary file 3 [file Data_Sheet_3.zip › Sit-miR319_Seita.6G158700.1_950_TPlot.pdf]

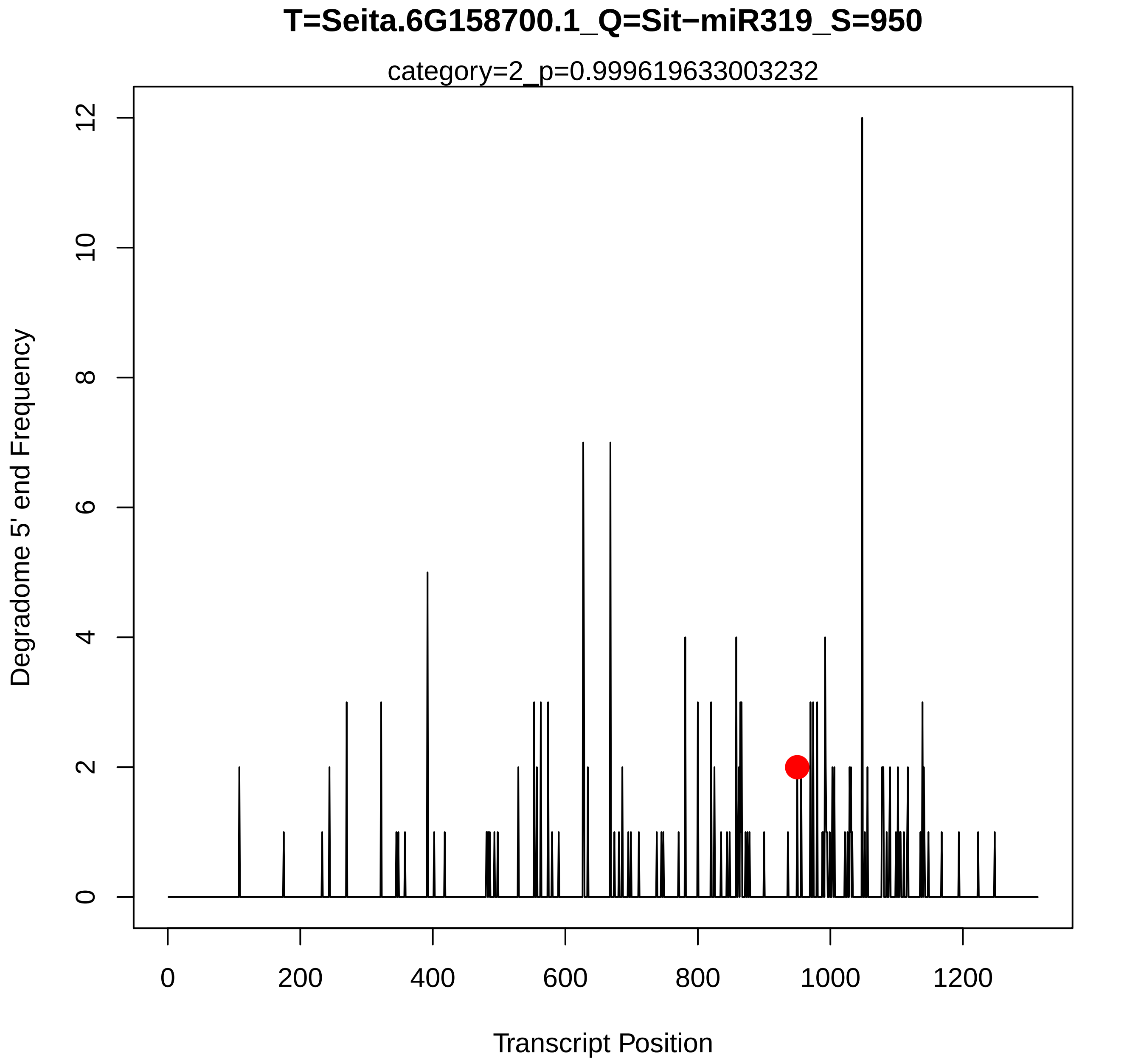

T=Seita.6G158700.1_Q=Sit−miR319_S=950
category=2_p=0.999619633003232
Degradome 5' end Frequency
0
2
4
6
8
10
12
0
200
400
600
800
1000
1200
Transcript Position
